# Antirheumatoid Arthritis Activities and Chemical Compositions of Phenolic Compounds-Rich Fraction from *Urtica atrichocaulis*, an Endemic Plant to China

**DOI:** 10.1155/2012/818230

**Published:** 2011-08-22

**Authors:** Mengyue Wang, Ke Li, Yuxiao Nie, Yingfang Wei, Xiaobo Li

**Affiliations:** ^1^School of Pharmacy, Shanghai Jiao Tong University, Shanghai 200240, China; ^2^School of Pharmacy, Chengdu University of Traditional Chinese Medicine, Chengdu 610075, China

## Abstract

*Urtica atrichocaulis*, an endemic plant to China, is commonly used to treat rheumatoid arthritis even though its pharmaceutical activities and chemical constituents were not studied. Herein, we reported our investigations on the chemical compositions of the phenolic compounds-rich fraction from *U. atrichocaulis* (TFUA) and their antirheumatoid arthritis activities. We found that the TFUA significantly inhibited the adjuvant-induced rats arthritis, carrageenin-induced rats paw edema, cotton pellet-induced mice granuloma, and the acetic acid-induced mice writhing response. Our phytochemical investigations on the TFUA resulted in the first-time isolation and identification of 17 phenolic constituents and a bis (5-formylfurfuryl) ether. The extensive HPLC analysis also revealed the chemical compositions of TFUA. Our further biological evaluation of the main phenolic components, individually and collectively, indicated that the antirheumatoid arthritis activities of TFUA were the combined effect of multiple phenolic constituents.

## 1. Introduction

 Rheumatoid arthritis is an immune-related inflammation disorder that affects over 60 million adults in the world wide. Its predominant symptoms include pain, stiffness, and swelling of peripheral joints. Rheumatoid arthritis may rapidly progress into a multisystem inflammation with irreversible joint destruction and increase the risk of mortality. However, the current treatment medications, such as nonsteroidal anti-inflammatory drugs, glucocorticosteroid, and immune-depressant are limited for their efficacy and frequently toxicity. Therefore, more and more patients look for medical botany options in coping with this debilitating disease. The recent investigation indicated that an estimated 60–90% of patients with rheumatoid arthritis were very likely to use botanicals. This growing interest in alternative medical practices clearly indicates the need for more safety and effective botanicals [1]. 

The genus Urtica (Urticaceae Family) is about 30 species and widely distributes in north and south temperate regions, also in mountainous areas of tropics [2]. Most species of genus Urtica are widely used for rheumatism and rheumatoid arthritis. *Urtica dioica *L. and *U. urens *L. have been used for arthritis for more than three thousand years in Europe [3]. In Asia, some Urtica species, such as *U. fissa* E. Pritz., *U. laetevirens *Maxim., and* U. dentata* (Hand.-Mazz.) C. J. Chen, are important herbs for rheumatism and rheumatoid arthritis [4]. The modern research showed that the aqueous extract of *U. laetevirens* Maxim. and* U. fissa* E. Pritz. had the potent inhibition of rheumatoid arthritis [5–7]. The ethanol extract of *U. dioica* L. significantly inhibited the proinflammatory transcription factor NF-*κ*B and the maturation of human myeloid dendritic cells, indicating that it possessed the therapeutic effect on T-cell-mediated inflammatory diseases like rheumatoid arthritis [8, 9]. The aqueous extract of *U. macrorrhiza *Hand.-Mazz. was reported to obviously suppress the PGE_2_ release from PM*φ* in adjuvant-induced arthritis rats and the expression of cyclooxygenase-2 (COX-2) mRNA induced by lipopolysaccharide [10]. 

*Urtica atrichocaulis* (Hand.-Mazz.) C. J. Chen is endemic to China and widely distributed in Guizhou, Sichuan, and Yunnan provinces [2]. This plant is a perennial herb, up to 150 cm tall, inflorescence containing proximal female flowers and distal male flowers, achene ovoid, smooth, up to 0.8 mm. In traditional chinese medicine, the aerial part of this plant is a commonly used herb for rheumatoid arthritis, with a history more than 1000 years. However, its chemical constituent and pharmacological activity have not been reported yet. We evaluated the antirheumatoid arthritis activities of different fractions and found that the phenolic compounds-rich fraction from *U. atrichocaulis* (TFUA) had the potent activity. In the subsequent investigation of chemical compositions of TFUA, 18 compounds, including 17 phenolic constituents, were isolated. The pharmacological activities of the main phenolic constituents were evaluated by adjuvant-induced rats arthritis model. The results suggested that the multiple phenolic constituents were responsible for the antirheumatoid arthritis activities of TFUA.

## 2. Material and Methods

### 2.1. Plant Material and Chemical Agents

*Urtica atrichocaulis* was collected from Xide County, Sichuan province (China) in April 2006, and authenticated by one of authors Professor Xiaobo Li. A voucher specimen (SJTU 20060419) was deposited in School of Pharmacy, Shanghai Jiao Tong University.

 Dexamethasone, indometacin, aspirin, morphine, and CMC were purchased from Shanghai National Pharmaceutical Co., Ltd. (Shanghai, China). Salicylic acid, phaselic acid, chlorogenic acid, and rutin for pharmacological test were purchased from Shanghai Ronghe Pharmaceutical Technology Development Co., Ltd. (Shanghai, China). Acetonitrile was HPLC grade from Shanghai Xingke Biochemistry Co., Ltd (Shanghai, China). Carrageenin (type IV) and Freund's complete adjuvant agent (FCA) were obtained from Sigma-Aldrich Corporation (Shanghai, China).

### 2.2. Experimental Animals

Kunming mice (18–22 g) and SD rats (180–220 g) were obtained from Shanghai Laboratory Animal Center of Chinese Academy of Sciences. They were acclimatized for 5 days before use and provided food and water *ad libitum*. The use of animals in this project was approved and in compliance with the regulations of the Institutional Animal Care and Use Committee of the School of Pharmacy, Shanghai Jiao Tong University, Shanghai, China.

### 2.3. Sample Preparation

The dried aerial part of *U. atrichocaulis *(7.7 kg) was extracted under reflux with alcohol 2 times for 2 h, filtrated, and concentrated in vacuum to give a greenish-black colored sticky extract (783 g). The extract (721 g) was suspended in 2000 mL water and sequently fractionated with petroleum ether (boiling range 30~60°C) and ethyl acetate. The ethyl acetate extract was evaporated and dried in vacuum to yield a yellowish-green extract, which was named the phenolic compounds-rich fraction from *U. atrichocaulis* (TFUA, 137 g). For pharmacological test, TFUA was dissolved in the 0.5% CMC solution.

### 2.4. Antirheumatoid Arthritis Activities

#### 2.4.1. Adjuvant-Induced Rats Arthritis

**Paw Swelling**

Adjuvant-induced rats arthritis was induced according to the method in the literature [11]. In brief, the right footpad of each male rat was injected subcutaneously with 0.1 mL FCA, containing 10 mg heat-inactive BCG in 1.0 mL paraffin oil. On the 8th day after FCA injection, rats were randomized in groups and orally given the sample, dexamethasone, and CMC solution daily for 15 consecutive days, respectively. The pad thicknesses of the left and right hind paws were measured before and every other day after FCA injection using a dial thickness gauge (Mitutoyo, Japan). The paw swelling at each time point was expressed as an increase in the footpad thickness (mm).

**Arthritis Index**

A blinded independent observer with no knowledge of the treatment protocol performed the arthritis index evaluation for each rat every other day from the 8th day after FCA injection. Each noninjected paw was graded separately from 0 to 4, depending on the severity. The assessment was made as follows: 0, no response; 1, slight edema of the digital joints; 2, edema of the digital joints and footpad; 3, gross edema of the entire footpad below the joint; 4, edema of the entire foot. In the case of the more severe responses, swellings of the tail and ears also were generally observed, but no additional score was ascribed for these signs. The arthritis index for each rat was the sum of the scores of the three measured joints [12, 13].

#### 2.4.2. Cotton Pellet Granuloma in Mice

Autoclaved cotton pellets weighing 5.0  ±  0.1 mg each were implanted subcutaneously through small incision made along the flank region of the male mice anesthetized with ether. The mice were orally administered the sample, dexamethasone, or CMC solution once daily for seven consecutive days from the day of cotton pellet insertion. On the 7th day, the mice were sacrificed and the cotton pellets covered by the granulomatous tissue were excised and dried in hot air oven at 60°C till a constant weight was achieved. Granuloma weight was obtained by subtracting the weight of cotton pellet on 0 d (before the start of experiment) from the weight of the cotton pellet on seventh day (at the end of experiment) [14].

#### 2.4.3. Carrageenin-Induced Rats Paw Edema

Male rats were treated orally with sample, indomethacin, and CMC solution 60 min prior to an injection of 0.1 mL of 1% carrageenin sterile (w/v) in saline into the plantar tissue of the right hind paw. The contralateral hind paws were injected with 0.1 mL of saline as control. Paw pad thickness was measured at 0, 2, 4 and 6 h after carrageenin injection, and paw swelling was obtained by subtracting the right pad thickness from the left pad thickness [12].

#### 2.4.4. Acetic Acid-Induced Mice Writhing Response

Mice were kept singly in a clear plastic observational cage (35 cm  ×  25 cm  ×  15 cm) and were pretreated with sample, aspirin or CMC solution by intragastric administration 60 min prior to intraperitoneal injection of 0.6% acetic acid in a volume of 0.1 mL per mouse. After the injection of acetic acid, the number of writhes exhibited for 15 min were counted. The percent inhibition was calculated as follows [15]:

(1) $$\begin{array}{c} \text{\%inhibition}=\frac{\text{mean}\;\;\text{writhes}\;\;\text{of}\;\;\text{vehicle}\;\;\text{group}-\text{mean}\;\;\text{writhes}\;\;\text{of}\;\;\text{test}\;\;\text{group}}{\text{mean}\;\text{writhes}\;\text{of}\;\text{vehicle}\;\text{group}}\times 100 \end{array}.$$

#### 2.4.5. Mice Hot-Plate Test

Female mice were placed on an YLS-6A intelligent hot-plate apparatus (Shandong Academy of Medical Sciences, China). The temperature of metal surface was maintained at 55.0  ±  0.5°C. Latency to a discomfort reaction (licking hind paw) was taken as pain threshold in mice and a cut-off time of 60 seconds was maintained to prevent scald. The fifty valid mice were selected (the pain threshold was determined in 5 to 30 seconds) and divided into five groups randomly. The deferent groups of mice were orally administered the sample, CMC solution, or intraperitoneally injected morphine. The pain threshold was determined before and 30, 60, 90, and 120 min after administration, respectively [16].

### 2.5. Statistical Analysis

Data were expressed as mean ± standard deviation (SD). Differences between groups were evaluated by Kruskal-Wallis test for the arthritic scores, or one-way analysis of variance (ANOVA) followed by Dunnett's test for the others indices with the aid of SPSS11.5 software package. Statistical significance is expressed as *P  <  0.05, **P  <  0.01.

## 3. Chemical Compositions Analysis

### 3.1. Isolation and Structure Identification of Compounds in TFUA

TFUA was fractionated by silica gel (200–300 mesh, Qingdao Haiyang chemical Co., Ltd.) column chromatography and gradually eluted with dichloromethane-methanol. The fractions obtained were further purified by repeat silica gel and Sephadex LH-20 (Pharmacia) column chromatography to yield pure compounds.

Compound identification was carried out by determination spectra data. The ^1^H (400 MHz) and ^13^C (100 MHz) NMR data were obtained with the solvent as reference, employing Bruker Avance 400 spectrometers. Molecular weight was obtained on Finnigan/MAT 4510 mass spectrometer. Optical rotation values were determined with a JASCO Dip-360 digital polarimeter.

### 3.2. Chemical Analysis of TFUA

Chemical analysis of TFUA was performed with an Agilent 1200 liquid chromatograph equipped with a DAD detector, quaternary pump, on-line degasser, autosampler, and thermostatic column compartment. Phenolic compounds were separated on an Agilent Zorbax Elipse XDB-C_18_ column (5 *μ*m, 250  ×  4.6 mm). Then the contents of main constituents in TFUA were calculated using their standards.

## 4. Results

### 4.1. Antirheumatoid Arthritis Effects of TFUA

TFUA showed the obvious therapeutic effect on the adjuvant-induced arthritis. It could significantly inhibit the paw swell induced by FCA and decreased the arthritis index, indicating it can inhibit the primary inflammation and second inflammation (Table 1). So its other pharmacological activities related to antirheumatoid arthritis, such as inhibiting nonspecific inflammation and analgesic activities, were warranted to the further evaluation.

**Figure 1:**
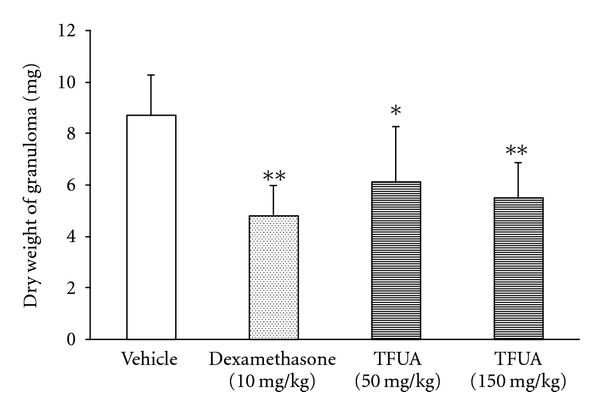
Effect of phenolic compounds-rich fraction from *Urtica atrichocaulis* (TFUA) on the weight of cotton pellet-induced granuloma in mice. Values are expressed as mean ± SD. *N*  =  10. *P  <  0.05, **P  <  0.01 compared with vehicle control.

The inhibiting activity of nonspecific inflammation was evaluated by the models of mice cotton pellet granuloma and carrageenin-induced rats paw edema. The results showed that TFUA had the moderate anti-inflammatory activity. It could inhibit the granuloma hyperplasia caused by cotton pellet at the dosages of 50 and 150 mg/kg (Figure 1). TFUA could reduce the carrageenin-induced rat paw edema also, and this activity became obvious at 4 h after administration (Table 2). 

The analgesic activity of TFUA was evaluated by the mice models of acetic acid-induced writhing response and hot-plate test. The results of acetic acid-induced writhing response revealed that TFUA had the obviously analgesic activity. It could significantly decrease the number of writhes induced by acetic acid (Table 3). However, the obvious analgesic activity of TFUA was not observed in the hot-plate test (Table 4). These results indicated that TFUA inhibited the inflammatory pain rather than neuropathic pain.

### 4.2. Isolation and Structure Identification of Compounds in TFUA

**Figure 2:**
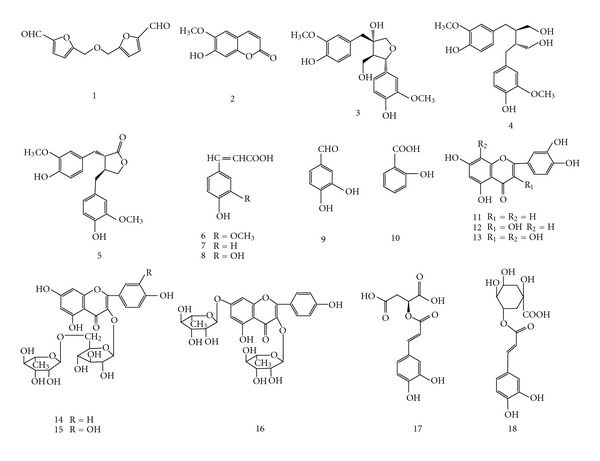
The chemical structures of 18 compounds isolated from TFUA. 1: bis (5-formylfurfuryl) ether; 2: scopoletin; 3: (–) olivil; 4: (–) secoisolariciresinol; 5: (–) matairesinol; 6: ferulic acid; 7: *p-*coumaric acid; 8: caffeic acid; 9: protocatechuic aldehyde; 10: salicylic acid; 11: luteolin; 12: quercetin; 13: gossypetin; 14: kaempferol-3-O-rutinoside; 15: rutin; 16: kaempferol 3, 7-di-O-rhamnopyranoside; 17: phaselic acid; 18: chlorogenic acid.

TFUA (83 g) was fractionated by silica gel column chromatography and gradually eluted with dichloromethane-methanol (95 : 5–60 : 40) to give 76 fractions of 500 mL each. These fractions were purified by repeat silica gel and sephadex LH-20 column chromatography and resulted in the isolation of 18 compounds (Figure 2). Their spectral data were as the following:

**Bis (5-formylfurfuryl) ether (1, 13 mg)**

Colorless needle (chloroform); HR EI-MS *m/z*: 234.0532, calcd for C_12_H_10_O_5_, 234.0528. ^1^H and ^13^C-NMR spectral data were consistent with those reported in the literature [17].

**Scopoletin (2, 8 mg)**

Colorless needle (MeOH). ^1^H and ^13^C-NMR spectral data were consistent with those reported in the literature [18].

**(–) Olivil (3, 6 mg)**

Colorless powder (MeOH); [*α*]_*D*_^20^−43.5° (*c*  =  1.0, CHCl_3_). ^1^H and ^13^C-NMR spectral data were consistent with those reported in the literature [19].

**(–) Secoisolariciresinol (4, 11 mg)**

Colorless powder (MeOH). [*α*]_*D*_^20^−21.3° (*c*  =  1.0, MeOH). ^1^H-NMR (CD_3_OD, 400 MHz) *δ*: 6.64 (2H, d, *J*  =  1.8 Hz, H-2, 2′), 6.69 (2H, d, *J*  =  8.0 Hz, H-5, 5′), 6.58 (2H, dd, *J*  =  1.8, 8.0 Hz, H-6, 6′), 2.67 (2H, dd, *J*  =  7.0, 13.8 Hz, H-7a, 7′a), 2.59 (2H, dd, *J*  =  7.0, 13. 8 Hz, H-7b, 7′b), 1.92 (2H, m, H-8, 8′), 3.61 (2H, m, H-9, 9′), 3.75 (6H, s, 2  ×  OCH_3_); ^13^C NMR (CD_3_OD, 100 MHz) *δ*: 134.2 (C-1, 1′), 114.0 (C-2, 2′), 149.1 (C-3, 3′), 145.7 (C-4, 4′), 116.0 (C-5, 5′), 123.0 (C-6, 6′), 36.3 (C-7, 7′), 44.5 (C-8, 8′), 62.4 (C-9, 9′), 56.1 (2  ×  OCH_3_) [20].

**(–) Matairesinol (5, 8 mg)**

Colorless powder (MeOH). [*α*]_D_^20^−37.0° (*c  *=  1.0, CHCl_3_). ^1^H and ^13^C-NMR spectral data were consistent with those reported in the literature [21].

**Ferulic Acid (6, 5 mg)**

Colorless styloid (MeOH); EI-MS *m/z*: 194 (M^+^); ^1^H and ^13^C-NMR spectral data were consistent with those reported in the literature [22].

***p-*Coumaric Acid (7, 10 mg)**

Colorless needle (acetone); ^1^H and ^13^C-NMR spectral data were consistent with those reported in the literature [22].

**Caffeic Acid (8, 7 mg)**

Yellowish needle (MeOH). ^1^H and ^13^C-NMR spectral data were consistent with those reported in the literature [23].

**Protocatechuic Aldehyde (9, 14 mg)**

Colorless needle (MeOH); ^1^H and ^13^C-NMR spectral data were consistent with those reported in the literature [24].

**Salicylic Acid (10, 33 mg)**

Colorless needle (MeOH); ^1^H and ^13^C-NMR spectral data were consistent with those reported in the literature [25].

**Luteolin (11, 6 mg)**

Yellow needle (MeOH); ^1^H and ^13^C-NMR spectral data were consistent with those reported in the literature [26].

**Quercetin (12, 15 mg)**

Yellow needle (MeOH); ^1^H and ^13^C-NMR spectral data were consistent with those reported in the literature [18].

**Gossypetin (13, 6 mg)**

Yellow needle (MeOH); ^1^H and ^13^C-NMR spectral data were consistent with those reported in the literature [27].

**Kaempferol-3-O-rutinoside (14, 23 mg)**

Yellow granule (MeOH); ^1^H and ^13^C-NMR (CDCl_3_, 100 MHz) spectral data were consistent with those reported in the literature [28].

**Rutin (15, 57 mg)**

Yellow powder (MeOH); ^1^H and ^13^C-NMR spectral data agreed with those reported in the literature [29].

**Kaempferol 3, 7-di-*O*-rhamnopyranoside (16, 10 mg)**

Yellow needle (MeOH); ^1^H-NMR (CD_3_OD, 400 MHz) *δ*: 7.80 (2H, d, *J*  =  8.7 Hz, H-2′, 6′), 6.94 (2H, d, *J*  =  8.7 Hz, H-3′, 5′), 6.75 (1H, d, *J*  =  2.0 Hz, H-8), 6.48 (1H, d, *J*  =  2.0 Hz, H-6), 5.06 (1H, d, *J*  =  2.0 Hz, 3-rha H-1), 5.04 (1H, d, *J*  =  2.0 Hz, 7-rha H-1), 1.25 (3H, d,* J  *=  6.1 Hz, 7-rha-Me), 0.93 (3H, d, *J*  =  5.5 Hz, 3-rha -Me); ^13^C-NMR (CD_3_OD, 100 MHz) *δ*: 158.1 (C-2), 136.5 (C-3), 179.8 (C-4), 163.1 (C-5), 99.9 (C-6), 163.6 (C-7), 95.6 (C-8), 161.1 (C-9), 107.6 (C-10), 122.4 (C-1′), 132.0 (C-2′, 6′), 116.6 (C-3′, 5′), 161.8 (C-4′). 3-rha: 103.5 (C-1), 72.1 (C-2), 73.2 (C-3), 73.6 (C-4), 71.9 (C-5), 18.1 (C-6); 7-rha: 100.6 (C-1), 71.7 (C-2), 72.1 (C-3), 73.6 (C-4), 71.3 (C-5), 17.7 (C-6) [30].

**Phaselic Acid (17, 28 mg)**

Yellow needle (MeOH); [*α*]_*D*_^20^+34.8° (*c  *=  2.0, H_2_O). ^1^H-NMR (CD_3_OD, 400 MHz): 6.96 (1H, d, *J*  =  8.0 Hz, H-5), 7.14 (1H, brd, *J*  =  8.0 Hz, H-6), 7.22 (1H, brs, H-2), 7.68 (1H, d, *J*  =  16.0 Hz, H-7), 6.44 (1H, d, *J*  =  16.0 Hz, H-8), 5.16 (1H, dd, *J*  =  2.8, 11.2 Hz, H-2′), 2.84 (1H, dd, *J*  =  2.8, 15.6 Hz, H-3′a), 2.65 (1H, dd, *J*  =  11.2, 15.6 Hz, H-3′b); ^13^C-NMR (CD_3_OD, 100 MHz): 147.6 (C-1), 146.7 (C-7), 144.6 (C-2), 127.8 (C-5), 123.4 (C-4), 116.8 (C-8), 115.7 (C-3), 115.1 (C-6), 178.8 (C-1′), 74.2 ( C-2′), 40.2 (C-3′), 178.0 (C-4′) [31].

**Chlorogenic Acid (18, 33 mg)**

Colorless needle (MeOH); ^1^H and ^13^C-NMR spectral data agreed with those reported in the literature [32].

### 4.3. HPLC Analysis of TFUA

**Figure 3:**
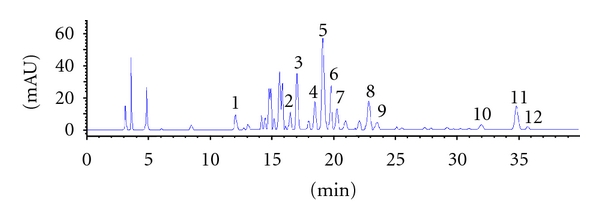
HPLC profile of phenolic compounds-rich fraction from *Urtica atrichocaulis* (TFUA). The concentration of TFUA was 1.0 mg/mL and injection volume was 10 *μ*L. The detection wavelength was set at 280 nm and the column was thermostated at 30°C. The eluent was H_2_O (containing 0.1% acetic acid)/acetonitrile. A linear gradient solvent system was used, starting from 95% H_2_O to 100% acetonitrile during a 40-min period, at the flow rate of 0.8 mL/min. 1: protocatechuic aldehyde; 2: caffeic acid; 3: chlorogenic acid; 4: secoisolariciresinol; 5: rutin; 6: phaselic acid; 7: kaempferol-3-O-rutinoside + kaempferol 3, 7-di-O-rhamnopyranoside; 8: salicylic acid; 9: scopoletin; 10: ferulic acid + gossypetin; 11: quercetin; 12: luteolin.

The chemical compositions of TFUA were further investigated by HPLC analysis. The results showed that phenolic acids and flavonoids were the main constituents. The relatively rich phenolics (contents above 5 percent) included rutin, chlorogenic acid, phaselic acid, and salicylic acid (Figure 3, Table 5).

### 4.4. The Antirheumatoid Arthritis Activities of the Main Phenolic Compounds and Their Combination

In order to screen the antirheumatoid arthritis constituents in TFUA, the pharmacological activities of the main phenolic acids, flavanoids, and their combination (the ratio was based on their contents in TFUA) were evaluated by FCA-induced rats arthritis model. The phenolic acids exhibited the statistically significant inhibition of edema in the injected paw (primary response) at dose levels of 50 mg/kg orally, when compared with the vehicle control group. Rutin showed moderate activity of inhibition of edema in the uninjected paw (second response). It is noted that neither phenolic acids nor flavanoids exhibited the significant suppressive effect on both primary swelling and second pathological changes of the experimental rats. However, their combination showed the obvious inhibition activity of paw edema in both the injected paw and the uninjected paw (Table 6). So, the antirheumatoid arthritis activity of TFUA may be the combined action of phenolic acids and flavanoids.

## 5. Discussion

It is well known that salicylic acid and its derivative aspirin had the anti-inflammatory and analgesic activity and often used to cure rheumatoid arthritis. However, these constituents only inhibit primary response, do not affect the second response and immune function, which is similar with the result of the present study. Although immunosuppressive agent and glucocorticosteroid inhibit secondary process, they have obvious side effect and toxicity. So the exploit of the antirheumatoid arthritis agent which could inhibit both the primary and secondary process, and possess good effect and lower toxicity from medical plant, had drawn much attention. 

*Urtica atrichocaulis* has been used as antirheumatoid herb in China for a long time and no obvious toxicity has been reported. In order to screen for its antirheumatoid arthritis fraction, the anti-inflammatory activity of three partition fractions, that is, petroleum ether fraction, ethyl acetate fraction, and water layer fraction from alcohol extract were evaluated by the adjuvant-induced rat arthritis model. The results showed that TFUA inhibited both primary and secondary processes and showed the potent activity of anti-nonspecific inflammation and analgesic activities (Table 1); however, the petroleum ether fraction and water layer fraction did not show the obvious activity (data not given). So the chemical compositions of TFUA were warranted to the systemic investigation.

In order to elucidate the constituents in TFUA, the systemic isolation of TFUA was carried out. As a result, eighteen compounds were isolated, of which the phenolic acids and flavanoids were the main constituents (Figure 3). Subsequently, the contents of the main constituents of TFUA were determined by HPLC. The result showed that chlorogenic acid, phaselic acid, rutin, and salicylic acid were the main phenolic constituents (Figure 3, Table 5).

In order to investigate the active constituents of TFUA, the antirheumatoid arthritis actions of main phenolic constituents were further evaluated. The results suggested that the main phenolic acids (chlorogenic acid, phaselic acid, and salicylic acid) in TFUA inhibited the primary response; the main flavanoids (rutin) inhibited the second response. The antirheumatoid arthritis of TFUA may be the combined action of multiple phenolic constituents.

Some phenolics were reported to possess the therapeutic effect on rheumatoid arthritis. Salicylic acid and its derivates had the potent anti-inflammatory activity and analgesic activity and usually used for rheumatoid arthritis patients to relieve the joint inflammation and pain. Recent research reported that scopoletin had good analgesic activity [33]; quercetin, rutin, and kaempferol-3-O-rutinoside had the immune modulation and antirheumatoid arthritis action, they reduce adjuvant-induced arthritis clinical signs and decrease human macrophage-derived inflammatory mediators [34–36]; kaempherol-3-O-rutinoside had the immunostimulatory activity and could possibly be useful for treating patients suffering from chronic granulomatous diseases [37]. Caffeic acid showed the powerful inhibitory effects on osteoclastogenesis and significantly suppressed expression of NFATc1 in adjuvant-induced arthritis rats [38]. The present study revealed that TFUA contained a high level of rutin, salicylic acid, caffeic acid, scopoletin, and quercetin. So, the antirheumatoid arthritis activity of *U. atrichocaulis* is very likely to be the combined action of multiple phenolic compounds.

## 6. Conclusions

In conclusion, the phenolic compounds-rich fraction from *Urtica atrichocaulis* inhibit the experimental arthritis induced by FCA and its activity may be the combined effect of multiple phenolic compounds. The antirheumatoid arthritis effects observed *in vivo *may give an explanation for the clinical application of *U. atrichocaulis* in therapy of rheumatoid diseases.

## Figures and Tables

**Table 1:** Effect of phenolic compounds-rich fraction from *Urtica atrichocaulis* (TFUA) on FCA-induced rats arthritis.

Group	Dose (mg/kg)	Paw swell (mm)						Arthritis index		
		12 d		16 d		20 d		12 d	16 d	20 d
		R	L	R	L	R	L			
Vehicle	—	4.21 ± 0.84	3.52 ± 1.01	3.86 ± 0.72	3.63 ± 0.71	3.47 ± 0.45	3.29 ± 0.73	7.6 ± 2.1	9.9 ± 2.6	10.3 ± 1.9
Dexamethasone	30	3.34 ± 0.66*	2.61 ± 0.76*	3.09 ± 0.56*	2.52 ± 0.76**	2.88 ± 0.27**	2.07 ± 0.62**	5.2 ± 2.3*	4.4 ± 1.8**	3.5 ± 2.7**
TFUA	50	3.77 ± 0.75	3.07 ± 0.94	3.61 ± 0.62	2.82 ± 0.97*	3.02 ± 0.46*	2.52 ± 0.56*	7.1 ± 1.9	8.7 ± 1.5	8.4 ± 2.3
	150	3.51 ± 0.61*	2.89 ± 0.85*	3.20 ± 0.67*	2.77 ± 0.85*	2.81 ± 0.61*	2.27 ± 0.68**	6.5 ± 2.0	7.8 ± 1.5*	7.6 ± 2.2*

Values are expressed as mean ± SD. *N  *=  10. *P  <  0.05, **P  <  0.01 compared with vehicle control.

**Table 2:** Effect of phenolic compounds-rich fraction from *Urtica atrichocaulis* (TFUA) on rats paw edema induced by carrageenin.

Group	Dose (mg/kg)	Paw swell (mm)		
		2 h	4 h	6 h
Vehicle	—	4.72 ± 0.88	5.15 ± 1.06	4.93 ± 0.92
Indometacin	30	3.78 ± 0.93*	3.22 ± 0.73**	2.37 ± 0.95**
TFUA	50	4.42 ± 1.26	4.08 ± 0.95*	3.69 ± 0.71*
	150	4.38 ± 0.65	3.64 ± 0.78**	3.20 ± 0.83**

Values are expressed as mean ± SD. *N*  =  10. *P  <  0.05, **P  <  0.01 compared with vehicle control.

**Table 3:** Effect of phenolic compounds-rich fraction from *Urtica atrichocaulis* (TFUA) on the mice writhes induced by acetic acid.

Group	Dose (mg/kg)	Number of writhes in 15 min	Inhibition rate (%)
Vehicle	—	29.5 ± 8.4	—
Aspirin	150	14.3 ± 4.8**	51.5
TFUA	50	20.5 ± 6.4**	30.5
	150	11.7 ± 3.2**	63.4

Values are expressed as mean ± SD. *N*  =  10. *P  <  0.05, **P  <  0.01 compared with vehicle control.

**Table 4:** Effect of phenolic compounds-rich fraction from *Urtica atrichocaulis* (TFUA) on the mice pain induced by hot-plate.

Group	Dose (mg/kg)	Anterior pain threshold (s)	Posterior pain threshold (s)			
			30	60	90	120
Vehicle	—	18.0 ± 3.5	20.1 ± 5.7	21.5 ± 5.6	21.2 ± 6.7	19.9 ± 8.1
Morphine	30	18.5 ± 3.5	30.4** ± 3.8	35.0** ± 6.0	28.6** ± 4.1	27.4** ± 4.6
TFUA	50	19.4 ± 3.8	21.6 ± 5.5	23.2 ± 6.1	21.7 ± 7.2	21.3 ± 8.8
	150	19.0 ± 3.7	21.2 ± 5.4	22.7 ± 5.9	21.4 ± 7.0	22.0 ± 8.5

Values are expressed as mean ± SD. *N*  =  10. *P  <  0.05, **P  <  0.01 compared with vehicle control.

**Table 5:** The content determination results of phenolic compounds-rich fraction from *Urtica atrichocaulis* (TFUA) by HPLC analysis (mg/g).

Phenolic acids		Flavonoids		The others	
Chlorogenic acid	88.3	Rutin	124.1	Secoisolariciresinol	37.5
Phaselic acid	62.4	Quercetin	30.9	Scopoletin	21.6
Salicylic acid	52.9	Luteolin	4.4		
Caffeic acid	17.6				
Protocatechuic aldehyde	10.3				

**Table 6:** The antirheumatoid arthritis activities of the main phenolic compounds and their combination.

Group	Dose (mg/kg)	Paw swell (mm)						Arthritis index		
		12 d		16 d		20 d		12 d	16 d	20 d
		R	L	R	L	R	L			
Vehicle	—	4.45 ± 0.93	3.80 ± 0.61	4.16 ± 0.89	4.03 ± 0.77	3.89 ± 0.54	3.57 ± 0.61	8.5 ± 2.3	10.8 ± 1.7	11.1 ± 2.4
Dexamethasone	30	3.33 ± 0.65*	3.09 ± 0.87*	2.92 ± 0.72**	2.84 ± 0.47**	2.23 ± 0.42**	2.24 ± 0.58**	6.2 ± 1.7*	4.9 ± 1.8**	3.7 ± 2.7**
Salicylic acid	50	3.57 ± 0.89*	3.67 ± 0.79	3.34 ± 0.56*	3.51 ± 0.83	2.95 ± 0.61*	3.25 ± 0.43	7.7 ± 2.8	10.1 ± 2.5	9.8 ± 1.7
Phaselic acid	50	3.88 ± 0.89	3.52 ± 0.85	3.09 ± 0.67**	3.62 ± 0.53	2.71 ± 0.49**	3.28 ± 0.73	6.9 ± 2.6	9.8 ± 1.7	10.4 ± 2.1
Chlorogenic acid	50	4.21 ± 0.66	3.55 ± 0.44	3.73 ± 0.75	3.66 ± 0.59	3.56 ± 0.85	3.39 ± 0.76	8.1 ± 2.5	10.3 ± 1.7	10.8 ± 2.3
Rutin	50	4.36 ± 0.80	3.40 ± 0.67	3.92 ± 0.54	3.29 ± 0.47*	3.78 ± 0.92	2.83 ± 0.87*	7.6 ± 2.0	9.3 ± 1.3	8.7 ± 1.9*
Combination^a^	50	3.48 ± 0.72*	3.21 ± 0.58*	3.40 ± 0.71*	3.04 ± 0.55**	2.84 ± 0.65**	2.61 ± 0.77**	7.2 ± 1.8	9.0 ± 1.9*	8.2 ± 1.5*

Values are expressed as mean ± SD. *N*  =  10. *P  <  0.05, **P  <  0.01 compared with vehicle control. ^a^The ratio of rutin  :  chlorogenic acid  :  phaselic acid  :  salicylic acid in combination was 12 : 9 : 6 : 5, according to their contents in phenolic compounds-rich fraction from *Urtica atrichocaulis* (TFUA).
